# Immunosuppressive therapy influences the accelerated age-dependent T-helper cell differentiation in systemic lupus erythematosus remission patients

**DOI:** 10.1186/s13075-018-1778-6

**Published:** 2018-12-18

**Authors:** Matthias Schaier, Claudius Gottschalk, Lorenz Uhlmann, Claudius Speer, Florian Kälble, Volker Eckstein, Carsten Müller-Tidow, Stefan Meuer, Karsten Mahnke, Hanns-Martin Lorenz, Martin Zeier, Andrea Steinborn

**Affiliations:** 1 Department of Medicine I (Nephrology), University of Heidelberg, Heidelberg, Germany; 2 Department of Obstetrics and Gynaecology, University of Heidelberg, Research Cooperation Unit Gynaecology/Nephrology, INF 162, 69120 Heidelberg, Germany; 3 Institute of Medical Biometry and Informatics, University of Heidelberg, Heidelberg, Germany; 4 Department of Medicine V (Haematology, Rheumatology), University of Heidelberg, Heidelberg, Germany; 5 Institute of Immunology, University of Heidelberg, Heidelberg, Germany; 6 Department of Dermatology, University of Heidelberg, Heidelberg, Germany

**Keywords:** Systemic lupus erythematosus, T-helper cell differentiation, Regulatory T cells, Proliferation capacity, Immunosuppressive therapy

## Abstract

**Background:**

CD4^+^ T cells are of great importance in the pathogenesis of systemic lupus erythematosus (SLE), as an imbalance between CD4^+^ regulatory T cells (Tregs) and CD4^+^ responder T cells (Tresps) causes flares of active disease in SLE patients. In this study, we aimed to find the role of aberrant Treg/Tresp cell differentiation for maintaining Treg/Tresp cell balance and Treg functionality.

**Methods:**

To determine differences in the differentiation of Tregs/Tresps we calculated the percentages of CD45RA^+^CD31^+^ recent thymic emigrant (RTE) Tregs/Tresps and CD45RA^+^CD31^−^ mature naive (MN) Tregs/Tresps, as well as CD45RA^−^CD31^+^ and CD45RA^−^CD31^−^ memory Tregs/Tresps (CD31^+^ and CD31^−^ memory Tregs/Tresps) within the total Treg/Tresp pool of 78 SLE remission patients compared with 94 healthy controls of different ages. The proliferation capacity of each Treg/Tresp subset was determined by staining the cells with anti-Ki67 monoclonal antibodies. Differences in the autologous or allogeneic Treg function between SLE remission patients and healthy controls were determined using suppression assays.

**Results:**

With age, we found an increased differentiation of RTE Tregs via CD31^+^ memory Tregs and of RTE Tresps via MN Tresps into CD31^−^ memory Tregs/Tresp in healthy volunteers. This opposite differentiation of RTE Tregs and Tresps was associated with an age-dependent increase in the suppressive activity of both naive and memory Tregs. SLE patients showed similar age-dependent Treg cell differentiation. However, in these patients RTE Tresps differentiated increasingly via CD31^+^ memory Tresps, whereby CD31^−^ memory Tresps arose that were much more difficult to inhibit for Tregs than those that emerged through differentiation via MN Tresps. Consequently, the increase in the suppressive activity of Tregs with age could not be maintained in SLE patients. Testing the Tregs of healthy volunteers and SLE patients with autologous and nonautologous Tresps revealed that the significantly decreased Treg function in SLE patients was not exclusively attributed to an age-dependent diminished sensitivity of the Tresps for Treg suppression. The immunosuppressive therapy reduced the accelerated age-dependent Tresp cell proliferation to normal levels, but simultaneously inhibited Treg cell proliferation below normal levels.

**Conclusions:**

Our data reveal that the currently used immunosuppressive therapy has a favorable effect on the differentiation and proliferation of Tresps but has a rather unfavorable effect on the proliferation of Tregs. Newer substances with more specific effects on the immune system would be desirable.

**Electronic supplementary material:**

The online version of this article (10.1186/s13075-018-1778-6) contains supplementary material, which is available to authorized users.

## Background

Systemic lupus erythematosus (SLE) is a chronic autoimmune disease with multiple clinical manifestations. It is characterized by periods of active disease and remission. The pathophysiology of SLE includes strong hyperactivity of B and T cells, resulting in exaggerated inflammation and the production of primarily nonorgan-specific autoantibodies towards antigens in the nucleus, in the cytoplasm, and on the cell surface [1]. Consequently, strong deposition of immune complexes and complement activation in blood vessels leads to progressive damage in different tissues, with lupus nephritis being the most common cause of morbidity and mortality [2]. The precise immune pathogenesis of SLE remains elusive. The production of autoantibodies by B cells and isotype switching depends on the help of autoreactive CD4^+^ T cells and on many other types of immune cells, such as dendritic cells, macrophages, and neutrophils [3]. Nevertheless, CD4^+^ T cells seem to be of greatest importance, as both SLE patients and lupus-prone mice show a characteristic activation of these cells, and inhibitors normalizing CD4^+^ T-cell reactions exhibit therapeutic effects.

Meanwhile, it is known that a fine balance between effector CD4^+^ T cells and immunosuppressive regulatory CD4^+^ T cells (Tregs) affects the immune homeostasis considerably, whereby the levels and functions of these cells were shown to be disturbed in autoimmune diseases [4]. Specifically, the interleukin (IL)-17, IL-21 and IL-22 producing T-helper (Th)17 cells, a lineage of effector CD4^+^ T cells, were found to contribute decisively to exaggerated inflammation and autoimmunity [5, 6]. A lot of data obtained in MLR/lpr and NZB/NZW mice suggest a key role of these cells in the pathogenesis of SLE; however, the therapeutic effects of Th17 cell antagonism for the treatment of SLE were found to be ineffective [7]. Since the expansion of Th17 cells has always been closely linked to the simultaneous exhaustion and dysfunction of Tregs, the development of therapeutics that regulate the imbalance between Th17 cells and Tregs was considered to be more promising than those that exclusively regulate Th17 cells [8]. A decisive effect of Tregs was shown to maintain self-tolerance by suppressing autoreactive T cells [9]. Therefore, the number and function of Tregs has been extensively studied in SLE patients, but the data available are very contradictory [10, 11]. These discrepancies can result from the use of different markers to characterize Tregs, whereby the combination of markers such as FoxP3 or CD25 with CD127 seems most suitable for characterizing Tregs in humans [12]. Moreover, the total Treg pool consists of different Treg subsets, which might differ functionally and could therefore contribute to the pathogenesis of SLE to varying degrees [13].

Furthermore, the differentiation of both Tregs and responder T cells (Tresps) or presumably of distinct subsets of Tregs and Tresps may be disturbed in SLE patients. Recent studies by our group have shown that special physiologic or pathologic conditions, such as old age, pregnancy, or even renal insufficiency in dialysis patients, may affect the distribution of CD45RA^+^CD31^+^ recent thymic emigrants (RTEs) from the thymus. Consequently, the already distributed RTE Tregs/Tresps differentiated more strongly into CD45RA^−^CD31^−^ memory Tregs/Tresps (CD31^−^ memory Tregs/Tresps). Thereby, the differentiation pathway via CD45RA^+^CD31^−^ mature naive (MN) Tregs/Tresps or CD45RA^−^CD31^+^ memory Tregs/Tresps (CD31^+^ memory Tregs/Tresps) influenced the strength of the suppressive activity of the Tregs on the proliferation of autologous Tresps considerably. We showed that differentiation via CD31^+^ memory Tregs increased the suppressive activity of the Tregs with age and particularly during pregnancy, while differentiation via MN Tregs caused decreased Treg function and loss of tolerance accompanied by the occurrence of pregnancy complications, such as preeclampsia and preterm labor [14–16]. In contrast, we found that the increased differentiation of RTE Tresps via MN Tresps into CD31^−^ memory Tresps with age in healthy individuals had been altered to an increased differentiation via CD31^+^ memory Tresps in old-aged dialysis patients, causing a decreased Treg functionality due to strengthened Tresp reactivity and chronic inflammation in these patients [17]. Such mismatched differentiation may cause the accumulation of senescent Tregs, thereby affecting the reactivity of the T-cell system substantially. Previous investigations already indicate that the immune system of patients with autoimmune diseases also show signs of accelerated aging [18].

For SLE patients, age-related changes in the immune system remain poorly understood. Therefore, we investigated whether increasing age had an effect on the proliferation, differentiation, and function of both Tregs and Tresps in healthy volunteers and SLE remission patients. With age, we found an increased differentiation of RTE Tregs via CD31^+^ memory Tregs and of RTE Tresps via MN Tresps into CD31^−^ memory Tregs/Tresp in healthy volunteers. This opposite differentiation of RTE Tregs and Tresps was associated with an age-dependent increase in the suppressive activity of both naive and memory Tregs. In contrast, SLE patients revealed an increased differentiation of the RTE Tresps via CD31^+^ memory Tresps instead of MN Tresps into CD31^−^ memory Tresps. Consequently, the increase in the suppressive activity with age could not be maintained in SLE patients. However, testing the Tregs of both healthy volunteers and SLE patients with both autologous and nonautologous Tresps revealed that the decreased Treg function was not exclusively attributed to an age-dependent diminished sensitivity of the Tresps for Treg suppression. The dual effect of the immunosuppressive medication on both Treg and Tresp cell proliferation also seemed to impair the autologous Treg functionality.

## Methods

### Patient collectives and healthy controls

Peripheral blood samples were collected from 94 healthy controls (mean age 45 ± 18 years, Group 1) and 78 SLE patients (mean age 45 ± 15 years, Group 2). All SLE patients fulfilled the 1983 revised and 1997 updated criteria of the American College of Rheumatology (ACR) for SLE [19, 20]. All SLE patients were diagnosed to be in remission (Systemic Lupus Erythematosus Disease Activity Index (SLEDAI) ≤ 4), where 56 patients (72%) had a documented period of renal manifestation within their disease history. Blood samples were collected during routine visits to the Department of Nephrology, University of Heidelberg.

### Fluorescence-activated cell sorting (FACS) staining

Peripheral venous blood samples (9 ml) were collected from all participants into EDTA-containing tubes. Whole peripheral blood mononuclear cells (PBMCs) were separated using Ficoll-Hypaque (inno-train Diagnostik GmbH, Kronberg, Germany) density gradient centrifugation, and subsequently analyzed with a six-color flow cytometer. Briefly, 8 × 10^6^ PBMCs were surface stained with 10 μl peridinin chlorophyll (PerCP)-conjugated anti-CD4 (BD Biosciences, Heidelberg, Germany), 5 μl phycoerythrin-cyanine 7 (PE-Cy7)-conjugated anti-CD127 (eBioscience, Frankfurt, Germany), 5 μl allophycocyanin-hilite 7 (APC-H7)-conjugated anti-CD45RA (BD Biosciences), and 5 μl Alexa-flour 647-conjugated anti-CD31 (BD Biosciences) mouse monoclonal antibodies. Intracellular staining for the detection of FoxP3 was performed using a fluorescein isothiocyanate (FITC)-conjugated anti-human FoxP3 staining set (clone PCH101, eBioscience) according to the manufacturer’s instructions. Detection of Ki67-positive cells within total CD4^+^ T cells, total Tregs/Tresps, and the different Treg/Tresp subsets was performed by incubating the fixed cells with 10 μl PE-conjugated anti-Ki67 monoclonal antibodies (clone B56, BD Biosciences). Negative control samples were incubated with isotype-matched antibodies. Forward- and side-scatter characteristics (FSC and SSC) were used to identify and exclude dead cells. Cells were analyzed by a FACS Canto flow cytometer (BD Biosciences). Statistical analysis was based on at least 100,000 CD4^+^ T cells.

### Positive selection of CD4^+^CD127^low+/–^CD25^+^ Tregs

To examine the suppressive activity of CD45RA^+^ naive Tregs and CD45RA^−^ memory Tregs, blood samples (50 ml) from 40 healthy controls and 37 SLE patients were collected in EDTA-containing tubes. Ficoll-Hypaque (inno-train Diagnostik GmbH) density gradient centrifugation was used to separate the PBMCs. The “Regulatory-T-cell Isolation Kit II” (Miltenyi Biotec, Bergisch Gladbach, Germany) was used to purify CD4^+^CD127^low+/–^CD25^+^ Tregs according to the manufacturer’s instructions. First, CD4^+^CD127^low+/−^ T cells were isolated by magnetic depletion of non-CD4^+^CD127^high+^ T cells. In the second step, the CD4^+^CD127^low+/–^CD25^+^ Tregs were isolated by positive selection over two consecutive columns. The CD4^+^CD127^low+/–^CD25^−^ T cells were obtained in the flow-through fraction and used as Tresps. The CD4^+^CD127^low+/–^CD25^+^ Tregs were subsequently retrieved from the columns.

### Sorting and functional testing of the different Treg subsets

For the sorting of the isolated CD4^+^CD127^low+/–^CD25^+^ Tregs into CD45RA^+^ naive Tregs and CD45RA^−^ memory Tregs, cells were stained with 5 μl PE-conjugated anti-CD45RA (BD Biosciences) and 5 μl APC-conjugated anti-CD45RO (eBioscience) mouse monoclonal antibodies. In all experiments, dead cells were excluded by FSC and SSC, while the remaining cells were sorted using a FACS Aria II or FACS Aria III cell sorter (BD Biosciences).

To analyze the suppressive activity of the isolated naive CD45RA^+^ Treg and CD45RA^−^ memory Treg populations, 2 × 10^4^ Tresps were cocultured with the purified Treg subsets at ratios of 1:2 to 1:1024 in 96-well v-bottom plates. Depending on the number of separated cells, the suppression assays were performed as single or multiple determinations. Suppression assays were performed in a final volume of 100 μl/well of X-VIVO15 medium (Lonza, Verviers, Belgium). For T-cell stimulation, the medium was supplemented with 1 μg/ml anti-CD3 and 2 μg/ml anti-CD28 antibodies (eBioscience, Frankfurt, Germany). As controls, CD4^+^CD127^low+/–^CD25^+^ Tregs and Tresps alone were cultured both with and without any stimulus. Cells were incubated at 37 °C in 5% CO_2_. After 4 days, 1 μCi ^3^H-thymidine (Hartmann Analytic, Braunschweig, Germany) was added to the cultures and cells were further incubated for 16 h. The cells were then harvested and ^3^H incorporation was measured by scintillation counting. To compare the suppressive activity of the different Treg subsets in healthy controls and SLE patients, the maximum suppressive activity (ratio of Tregs to Tresps 1:2) and the minimum ratio of Tregs to Tresps at which a suppression of at least 15% could be achieved were calculated [21].

For further examination of Treg and Tresp functionality, blood samples of 14 SLE patients and age-matched healthy controls were taken and processed as described above. The obtained naive CD45RA^+^ Tregs, CD45RA^−^ memory Tregs, and CD4^+^CD127^low+/–^CD25^−^ Tresps were arranged into a suppression assay in the same way as before, but both Treg populations from SLE patients were cocultured with Tresps from healthy controls and vice versa.

### Statistical analysis

Linear regression was used to evaluate the influence of age on the composition of total CD4^+^ T-helper cells with Tregs and Tresps for both healthy controls and SLE patients using separate models. The same approach was used for evaluating the changes with age in the composition of total Tregs/Tresps with their subsets (RTE, MN, CD31^+^memory, and CD31^−^ memory Tregs/Tresps) and for evaluating changes in the percentage of Ki67^+^ cells within total Tregs/Tresps and their subsets (RTE, MN, CD31^+^ memory, and CD31^−^ memory-Tregs/Tresps). In addition, we calculated the Pearson correlation coefficients (*r*) between age and the change in the variables. An analogous procedure was chosen for evaluating the changes in the percentages of RTE Tregs/Tresp and MN Tregs/Tresps within total naive CD45RA^+^ Tregs/Tresps with age or with their Ki67 expression. Differences between the two patient groups (healthy volunteers and SLE patients) concerning the above listed Treg/Tresp subsets were examined using multiple regression analysis adjusted for the age variable (centered on the mean), wherein an interaction term of the age and the patient group was included. Changes of the suppressive activity of the different Treg subsets (naive CD45RA^+^ Tregs, CD45RA^−^ memory Tregs) with age were also analyzed using linear regression. A *p* value < 0.05 was considered significant. For all tests, the software package BiAS for Windows (version 10.06) was used.

## Results

### SLE remission patients show reduced Treg proliferation but excessive Tresp proliferation

**Fig. 1:**
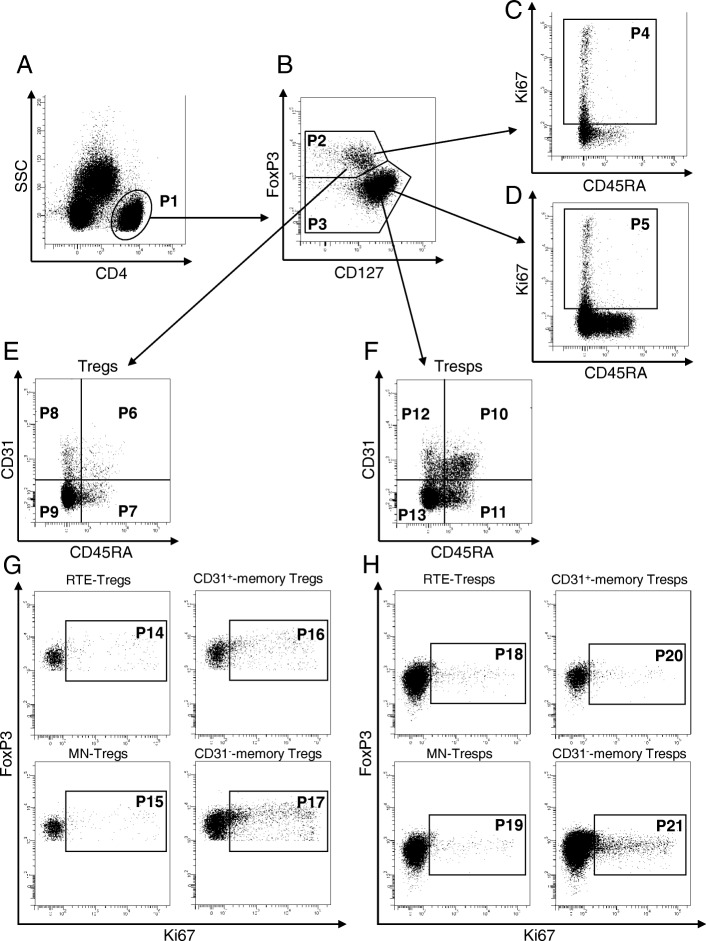
Gating strategy for six-color flow cytometric detection of recent thymic emigrant (RTE), mature naive (MN), CD31^+^, and CD31^−^ memory regulatory T cells (Tregs)/responder T cells (Tresps). At first, CD4^+^ T cells (P1) were gated by side scatter characteristics (SSC) versus fluorescence intensity of CD4 (**a**). Then CD4^+^CD127^low+/–^FoxP3^+^ Tregs (P2) and CD4^+^CD127^+^FoxP3^−^ Tresps (P3) were gated by fluorescence intensity of FoxP3 versus CD127 (**b**). Ki67^+^ cells of CD4^+^CD127^low+/–^FoxP3^+^ Tregs (P4) and CD4^+^CD127^+^FoxP3^−^ Tresps (P5) were gated by fluorescence activity of Ki67 versus CD45RA (**c** and **d**). The percentages of RTE Tregs/Tresps (P6, P10), MN Tregs/Tresps (P7, P11), CD31^+^ memory Tregs/Tresps (P8, P12), and CD31^−^ memory Tregs/Tresps (P9, P13) were estimated by analyzing the CD4^+^CD127^low+/–^FoxP3^+^ Treg pool (**e**) and the CD4^+^CD127^+^FoxP3^−^ Tresp pool (**f**) for its fluorescence intensity of CD31 versus CD45RA. The Ki67 expression of RTE Tregs/Tresps (P14, P18), MN Tregs/Tresps (P15, P19), CD31^+^ memory Tregs/Tresps (P16, P20), and CD31^−^ memory Treg/Tresps (P17, P21) were estimated by analyzing the fluorescence intensity of FoxP3 versus Ki67, respectively (**g** and **h**)

To examine whether there were age-dependent differences in the composition of the total CD4^+^ T-helper cell pool with total CD4^+^CD127^low+/–^FoxP3^+^ regulatory T cells (Tregs) and CD4^+^CD127^+^FoxP3^−^ responder T cells (Tresps), we estimated the percentages of both T-cell subsets in 78 SLE patients and 94 healthy controls of various ages. Furthermore, we determined the age-dependent proliferation capacity of Tregs and Tresps by measuring their percentages of Ki67^+^ cells and investigated whether there were differences in the differentiation of Tregs and Tresps between both study groups. For this, we calculated the percentages of RTE and MN Tregs/Tresps, as well as CD31^+^ and CD31^−^ memory Treg/Tresps within the total Treg/Tresp pool and determined the proliferation capacity of each Treg/Tresp subset. To identify differences in the age-dependent differentiation pathway of RTE Tregs/Tresps via MN Tregs/Tresps or CD31^+^ memory Tregs/Tresp into CD31^−^ memory Tregs/Tresps between healthy controls and SLE patients, we determined the percentages of RTE and MN Tregs/Tresps within the naive CD45RA^+^ Treg/Tresp pool with age and correlated these data with their proliferation capacity. To investigate the differentiation of resting naive MN Tregs/Tresps, we correlated their percentage within total CD31^−^ Tregs/Tresps with their Ki67 expression. Figure 1 shows the gating strategy that was used in all experiments and Table 1 presents the clinical data of all participants in this study.

**Table 1:** Clinical characteristics of SLE patients and healthy controls

	Healthy controls *n* = 94	SLE patients *n* = 78	SLE patients treated with glucocorticoids *n* = 46	SLE patients not treated with glucocorticoids *n* = 32	SLE patients treated with MMF or AZA *n* = 48	SLE patients not treated with MMF or AZA *n* = 30
Female sex, *n* (%)	64 (68%)	65 (83%)	40 (87%)	25 (78%)	38 (79%)	27 (90%)
Age (years)	45 ± 18	45 ± 15	47 ± 16	43 ± 12	43 ± 14	50 ± 16
Time since initial diagnosis (years)	–	14 ± 9	14 ± 9	12 ± 9	14 ± 8	14 ± 10
Renal involvement, *n* (%)	–	56 (72%)	38 (87%)*	18 (56%)*	38 (79%)	18 (60%)
Medication						
No medication, *n* (%)		4 (5%)	–	4 (13%)	–	4 (13%)
Antimalarials, *n* (%)		62 (79%)	36 (78%)	26 (81%)	40 (83%)	22 (73%)
Mycophenolate mofetil, *n* (%)		31 (40%)	19 (41%)	12 (38%)	31 (65%)	–
Azathioprine, *n* (%)		17 (22%)	9 (20%)	8 (25%)	17 (35%)	–
Glucocorticoids, *n* (%)		46 (59%)	46 (100%)	–	28 (58%)	18 (60%)
Glucocorticoid dose (mg/day)		3.93 ± 1.53	3.93 ± 1.53	–	3.89 ± 1.70	3.99 ± 1.27
Serum leukocytes (*n*/l)	–	6.65 ± 2.36	7.09 ± 2.36	6.02 ± 2.25	6.50 ± 2.55	6.90 ± 2.04
Serum creatinine (mg/dl)	–	0.97 ± 0.61	0.94 ± 0.60	1.00 ± 0.64	1.05 ± 0.74	0.84 ± 0.28
CKD-EPI GFR (ml/min/1.73 m^2^)	–	88.6 ± 31.0	88.3 ± 31.2	89.0 ± 31.3	88.0 ± 35.0	89.6 ± 23.9
MDRD GFR (ml/min/1.73 m^2^)	–	88.0 ± 32.8	87.2 ± 31.9	89.2 ± 34.6	87.9 ± 36.7	88.3 ± 26.0
Urine protein/urine creatinine ratio (g/mol creatinine)	–	44.8 ± 81.6	55.2 ± 98.8	29.7 ± 44.3	62.7 ± 98.8^†^	16.1 ± 21.5^†^

A *p* value < 0.05 was considered significant

The data are presented as their mean and standard deviation unless otherwise indicated

*AZA* azathioprine, *CKD-EPI GFR* Chronic Kidney Disease Epidemiology Collaboration-estimated glomerular filtration rate, *MDRD GFR* Modification of Diet in Renal Disease study-estimated glomerular filtration rate, *MMF* mycophenolate mofetil, *SLE* systemic lupus erythematosus

*^, †^ Significantly differing values between groups (nonparametric H test of Kruskal and Wallis, followed by a Dunn test)

**Fig. 2:**
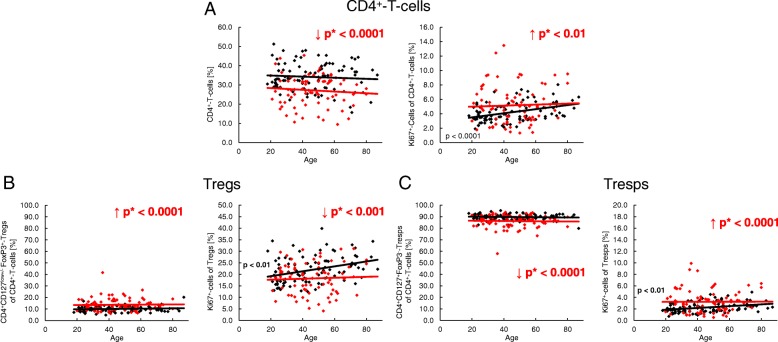
The proportion of Ki67^+^ cells of CD4^+^ T cells, total CD4^+^CD127^low+/–^FoxP3^+^ regulatory T cells (Tregs) and total CD4^+^CD127^+^FoxP3^−^ responder T cells (Tresps) during the life course in healthy volunteers (*n* = 94) and SLE patients (*n* = 78). The percentages of CD4^+^ T cells within PBMCs (**a**), and of total Tregs (**b**) or total Tresps (**c**) within the total CD4^+^ T-helper cell pool, as well as their Ki67 expression, are shown for healthy volunteers (black diamonds) and SLE patients (red diamonds) during the course of life. The figures present the regression lines concerning the changes in the percentages of the individual T-cell subsets together with their Ki67 expression with increasing age. Significant age-dependent changes in healthy volunteers are marked by black *p* values. Significant age-independent differences between healthy volunteers and SLE patients are marked by red arrows (red downward and upward arrows) and red p* values

We found that the percentage of CD4^+^ T-helper cells was significantly decreased in SLE patients, regardless of age. However, the proliferation capacity of CD4^+^ T-helper cells, which increased significantly with age in healthy controls, was significantly increased in SLE patients (Fig. 2a). In addition, the percentage of Tregs within the total CD4^+^ T-helper pool was strongly increased in these patients (Fig. 2b), while the Tresp pool was complementarily diminished (Fig. 2c). By measuring the percentage of Ki67^+^ cells, we noticed an age-dependent significant increase for both Tregs and Tresps in healthy volunteers which could not be detected in SLE patients (Fig. 2b, c). Compared with healthy volunteers, we found an age-independent significantly lower percentage of Ki67^+^ cells in Tregs (Fig. 2b), but a higher percentage of Ki67^+^ cells in Tresps (Fig. 2c) in SLE patients. Presumably, this means that there is an excessive Tresp cell proliferation in SLE patients which cannot be sufficiently suppressed by the immunosuppressive therapy.

### SLE remission patients show an increased differentiation of their Treg pool, but not of their Tresp pool

**Fig. 3:**
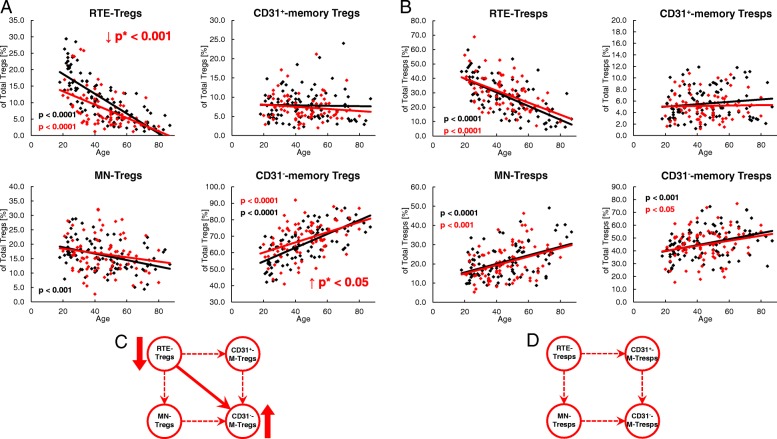
Changes in the composition of the total regulatory T cell (Treg)/responder T cell (Tresp) pool with age in healthy volunteers (*n* = 94) and SLE patients (*n* = 78). The percentages of recent thymic emigrant (RTE) Tregs/Tresps, mature naive (MN) Tregs/Tresps, CD31^+^ memory Tregs/Tresps, and CD31^−^ memory Tregs/Tresps were estimated within the total Treg/Tresp pool in both healthy volunteers (black diamonds) and SLE patients (red diamonds). The figures present the regression lines concerning the changes in the percentages of the different Treg/Tresp subsets with increasing age. Significant changes with age are marked by black *p* values (healthy volunteers) or red *p* values (SLE patients). Significantly decreased percentages (red downward arrow) of RTE Tregs, but increased percentages (red upward arrow) of CD31^−^ memory Tregs within total Tregs, independently of age (marked by red p* values), suggest an enhanced differentiation of RTE Tregs into CD31^−^ memory Tregs in SLE patients compared with healthy volunteers (**a**). Age-independent differences in the differentiation of RTE Tresps between healthy volunteers and SLE patients were not detected (**b**). The possible differentiation pathways of RTE Tregs/Tresps are illustrated by dashed arrows (**c** and **d**). The enhanced differentiation of RTE Tregs into CD31^−^ memory Tregs is illustrated by a bold arrow (**c**). Details regarding the enhanced differentiation pathway of RTE Tregs via MN or CD31^+^ memory Tregs in SLE patients cannot be determined (**d**)

To detect differences in the differentiation of Tregs/Tresps between healthy volunteers and SLE patients, we estimated the percentages of RTE and MN Tregs/Tresps, as well as CD31^+^ memory and CD31^−^ memory-Tregs/Tresps within the total Treg/Tresp pool with age in both study groups. During a healthy course of life, the percentages of RTE and MN Tregs decreased significantly, CD31^+^ memory Tregs did not change, and CD31^−^ memory Tregs increased significantly within total Tregs (Fig. 3a). A similar differentiation was revealed for Tresps, with the exception of MN Tresps which increased significantly with age (Fig. 3b). It became apparent that the age-dependent differentiation of Tregs and Tresps was not principally different between healthy controls and SLE patients. However, regardless of age, the percentages of RTE Tregs were significantly decreased, while the percentages of CD31^−^ memory Tregs were increased, indicating an enhanced differentiation of Tregs in SLE patients, particularly in young individuals (Fig. 3c). Concerning the differentiation of Tresps, we did not reveal any significant differences between both study groups (Fig. 3d). The determination of the different Treg/Tresp subsets within total CD4^+^ T cells also showed that there were no differences concerning the age-dependent differentiation of RTE Tregs/Tresps into CD31^−^ memory Tregs/Tresps between healthy controls and SLE patients. Age-independent evaluation of Treg/Tresp subsets within the total CD4^+^ T-cell pool also revealed no changes regarding Tresp differentiation. However, equal percentages of RTE Tregs, but significantly increased percentages of more differentiated Treg subsets (MN Tregs, CD31^+^ memory Tregs and CD31^−^ memory Tregs) were detected within total CD4^+^ T cells of SLE patients compared with healthy controls, proposing a differentiation which rather increases Tregs than Tresps in SLE patients (Additional file 1: Figure S1).

### SLE remission patients show an age-independent increased differentiation of RTE Tregs via CD31^+^ memory Tregs into CD31^−^ memory Tregs, while RTE Tresps differentiate more strongly via both CD31^+^ memory Tresps and MN Tresps

**Fig. 4:**
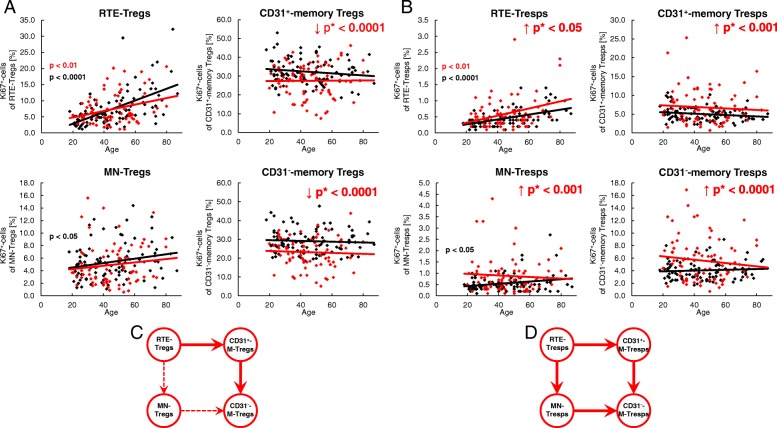
Changes in the Ki67 expression of recent thymic emigrant (RTE), mature naive (MN), CD31^+^, and CD31^−^ memory regulatory T cells (Tregs)/responder T cells (Tresps) with age in healthy volunteers (*n* = 94) and SLE patients (*n* = 78). The percentages of Ki67^+^ cells within RTE Tregs/Tresps, MN Tregs/Tresps, CD31^+^ memory Tregs/Tresps, and CD31^−^ memory Tregs/Tresps were determined in both healthy volunteers (black diamonds) and SLE patients (red diamonds) (**a** and **b**). The figures present the regression lines concerning the changes of Ki67 expression of the individual Treg/Tresp subsets with age. Significant changes with age are marked by black *p* values (healthy volunteers) or red *p* values (SLE patients). The age-independent significantly decreased (red downward arrow) percentage of Ki67^+^ cells within CD31^+^ memory Tregs and CD31^−^ memory Tregs (marked by red p* values) suggests an increased differentiation of the RTE Tregs via CD31^+^ memory Tregs into CD31^−^ memory Tregs in SLE patients (bold arrows), which is strongly suppressed by the immunosuppressive therapy (**a** and **c**). The age-independent significantly increased (red upward arrow) percentage of Ki67^+^ cells within all Tresp subsets (marked by red p* values) suggests an increased differentiation of the RTE Tresps via both MN and CD31^+^ memory Tresps into CD31^−^ memory Tresps in SLE patients (bold arrows) which is not completely suppressed by the immunosuppressive therapy (**b** and **d**)

To detect age-related differences in the proliferation of the different Treg/Tresp subsets, we estimated the percentages of Ki67^+^ cells in healthy volunteers and SLE patients of different ages. In healthy volunteers, the proliferation capacity increased significantly in RTE and MN Tregs/Tresps with age, but did not change in CD31^+^ or CD31^−^ memory Tregs/Tresps (Fig. 4a, b). In SLE patients, this age-dependent increase in the proliferation capacity was preserved in RTE Tregs/Tresps, and also largely for MN Tregs but not for MN Tresps (Fig. 4a, b). In addition, we observed an age-independent significantly decreased proliferation capacity of CD31^+^ and CD31^−^ memory Tregs, but a significantly increased proliferation capacity of all four Tresp subsets in these patients (Fig. 4a, b). This could be due to the effect of the immunosuppressive therapy reducing the proliferation capacity, especially of those Treg populations which show significantly increased proliferation. Consequently, our findings may suggest that, regardless of age, the RTE Tregs differentiate more strongly via CD31^+^ memory Tregs into CD31^−^ memory Tregs in SLE patients compared with healthy controls (Fig. 4c). In contrast, the age-independent significantly increased proliferation capacity detected in all Tresp subsets of SLE patients suggests that RTE Tresps differentiate more strongly via both CD31^+^ memory Tresps and MN Tresps into CD31^−^ memory Tresps (Fig. 4d). Presumably, in SLE patients the proliferation capacity of the RTE Tresps is severely increased compared with that of their RTE Tregs, so that the immunosuppressive therapy regulates the proliferation of the arising Treg subsets below the normal level; however, is not able to inhibit the proliferation of the arising Tresp subsets as effectively.

### Compared with healthy controls, SLE patients show differences concerning their age-dependent differentiation of Tresps, but not of their Tregs

**Fig. 5:**
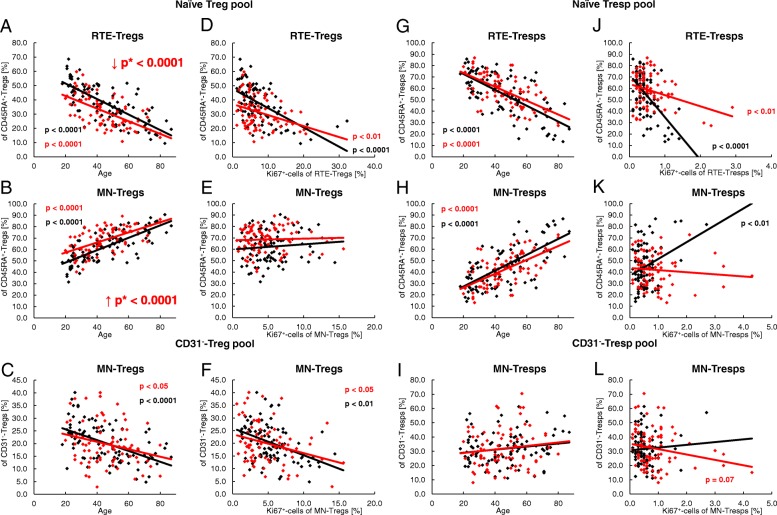
Changes in the composition of the naive CD45RA^+^ regulatory T cell (Treg)/responder T cell (Tresp) pool and the CD31^−^ Treg/Tresp pool during the course of life in healthy volunteers (*n* = 94) and SLE patients (*n* = 78). The percentages of recent thymic emigrant (RTE) and mature naive (MN) Tregs/Tresps within the naive CD45RA^+^ Treg/Tresp pool, as well as those of MN Tregs/Tresps within the total CD31^−^ Treg/Tresp pool, were estimated in healthy volunteers (black diamonds) and SLE patients (red diamonds). The figures present the regression lines concerning the changes of the Treg/Tresp subsets with age (**a**–**c** and **g**–**i**) or with their Ki67-expression (**d**–**f** and **j**–**l**). Significant changes with age are marked by black *p* values (healthy volunteers) or red *p* values (SLE patients). In SLE patients, age-independent significantly decreased (red downward arrow) percentages of RTE Tregs within the naive CD45RA^+^ Treg pool (**a**) but increased (red upward arrow) percentages of MNs within the naive CD45RA^+^ Treg pool (**b**) (marked by red p* values) were detected, but not for RTE Tresps or for MN Tresps within the total CD45RA^+^ Tresp pool (**g** and **h**). A significant negative correlation between the percentage of RTE Tregs/Tresps and their Ki67 expression was found for both healthy volunteers and SLE patients (**d** and **j**). A significant correlation between the percentage of MN Tregs within total naive CD45RA^+^ Tregs and their Ki67 expression was not found for healthy volunteers or for SLE patients (**e**). A significant positive correlation was found between the percentage of MN Tresps within total naive CD45RA^+^ Tresps and their Ki67 expression which could not be assessed for SLE patients (**k**). The percentage of resting naive MN Tregs within total CD31^−^ Tregs decreased significantly with age (**c**), while that of resting naive MN Tresps increased slightly, but not significantly, within total CD31^−^ Tresps (**i**). A significant negative correlation between the percentage of resting naive MN Tregs within total CD31^−^ Tregs and their Ki67 expression was found for both study groups (**f**). In contrast, the percentage of MN Tresps within CD31^−^ Tresps did not correlate with their Ki67 expression in healthy controls but showed a nearly significant negative correlation for SLE patients (**l**)

To further detect differences in the age-dependent differentiation between healthy volunteers and SLE patients, we examined the imminent differentiation of RTE Tregs/Tresps via MN Tregs/Tresps into CD31^−^ memory Tregs/Tresps, as well as the differentiation of resting naive MN Tregs/Tresps into CD31^−^ memory Tregs/Tresps. For this, we first determined the percentages of RTE and MN Tregs/Tresps within total naive CD45RA^+^ Tregs/Tresps, as well as the percentages of MN Tregs/Tresps within total CD31^−^ Tregs/Tresps with increasing age. Independent of age, we detected significantly lower percentages of RTE Tregs (Fig. 5a), but higher percentages of MN Tregs (Fig. 5b) within naive CD45RA^+^ Tregs in SLE patients, while the percentages of MN Tregs within CD31^−^ Tregs were not affected (Fig. 5c). With age, we found significantly decreasing percentages of RTE Tregs (Fig. 5a), but increasing percentages of MN Tregs (Fig. 5b) in the naive CD45RA^+^ Treg pool, associated with significantly decreasing percentages of MN Tregs in the CD31^−^ Treg pool for both study groups (Fig. 5c). Next, we correlated the percentages of RTE and MN Tregs within total naive CD45RA^+^ Tregs and their Ki67 expression. We found a significant negative correlation for the percentage of RTE Tregs and their Ki67 expression in both healthy volunteers and SLE patients (Fig. 5d). These findings suggest that decreasing percentages of RTE Tregs within the total naive CD45RA^+^ Treg pool are associated with an increased Ki67 expression of these cells, whereby their differentiation into either MN Tregs or CD31^+^ memory Tregs may be strengthened. Neither a significant negative or positive correlation was detected for MN Tregs within naive CD45RA^+^ Tregs and their Ki67 expression in both study groups (Fig. 5e). As RTE Tregs decrease within total naive Tregs with age, and MN Tregs increase, these findings suggest that RTE Tregs differentiate increasingly via CD31^+^ memory Tregs into CD31^−^ memory Tregs in both healthy volunteers and SLE patients. However, there was also a significant negative correlation for the percentage of MN Tregs within total CD31^−^ Tregs and their Ki67 expression in both study groups (Fig. 5f), indicating an age-dependent significantly increased conversion of resting naive MN Tregs in both healthy volunteers and SLE patients.

In contrast, we did not detect any significant age-independent differences in the composition of the naive CD45RA^+^ Tresp pool with RTE or MN Tresps between healthy volunteers and SLE patients (Fig. 5g, h). In addition, the percentage of MN Tresps within total CD31^−^ Tresps was not affected (Fig. 5i). With age, we found significantly decreasing percentages of RTE Tresps but increasing percentages of MN Tresps in both study groups (Fig. 5g, h), while the percentages of MN Tresps within total CD31^−^ Tresps increased slightly but not significantly (Fig. 5i). Both healthy volunteers and SLE patients showed a significant negative correlation between the percentage of RTE Tresps and their Ki67 expression indicating that, similar to RTE Tregs, RTE Tresps show an increased differentiation with increasing age (Fig. 5j). However, in healthy volunteers we found an additional positive correlation between the percentage of MN Tresps within total naive CD45RA^+^ Tresps and their Ki67 expression (Fig. 5k). These findings suggest that, with age, RTE Tresps of healthy volunteers differentiate increasingly via MN Tresps into CD31^−^ memory Tresps. As this correlation could not be detected for SLE patients (Fig. 5k), it can be assumed that, with age, the RTE Tresps of these patients differentiate increasingly via CD31^+^ memory Tresps into CD31^−^ memory Tresps. Neither a positive or negative correlation between the percentage of resting naive MN Tresps within total CD31^−^ Tresps and their Ki67 expression could be detected for healthy controls (Fig. 5l), indicating that there is no age-dependent increased conversion of resting naive MN Tresps into CD31^−^ memory Tresps. However, an almost significant negative correlation between the percentages of resting naive MN Tresps within CD31^−^ Tresps and their Ki67 expression for SLE patients suggests that there may be a generally increased conversion of these cells into CD31^−^ memory Tresps, independent of age (Fig. 5l).

### Corticosteroids inhibit the proliferation capacity of Tregs regardless of age

**Fig. 6:**
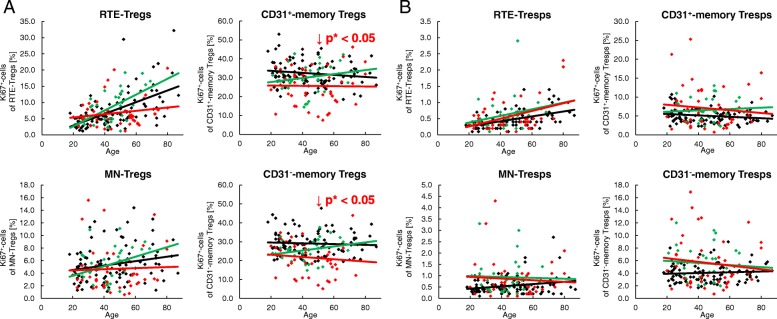
Changes of the Ki67 expression of recent thymic emigrant (RTE), mature naive (MN), CD31^+^, and CD31^−^ memory regulatory T cells (Tregs)/responder T cells (Tresps) with age in healthy volunteers (*n* = 94) and SLE patients, divided into patients treated with glucocorticoids (*n* = 46) or not (*n* = 32). The percentages of Ki67^+^ cells within RTE, MN, CD31^+^, and CD31^−^ memory Tregs/Tresp was estimated in healthy volunteers (black diamonds) and in SLE patients who were treated with glucocorticoids (red diamonds) or not (green diamonds). An age-independent significantly decreased (red downward arrow) percentage of Ki67^+^ cells was detected in CD31^+^ and CD31^−^ memory Tregs (marked by red p* values) in glucocorticoid-treated versus untreated SLE patients (**a**). A similarly reduced expression of Ki67^+^ cells in CD31^+^ and CD31^−^ memory Tresps of glucocorticoid-treated SLE patients was not detected (**b**)

For the treatment of SLE, different immunosuppressive drugs are used. Figure 6 shows the proliferation capacity of all four Treg/Tresp subsets depending on whether the patients were treated with corticosteroids (*n* = 46) or not (*n* = 32). These two patient groups differed clinically only in the fact that renal involvement was diagnosed significantly more often in patients treated with glucocorticoids (Table 1). These patients showed a significantly decreased age-independent proliferation capacity of their CD31^+^ and CD31^−^ memory Treg subsets compared with patients who had not received these drugs (Fig. 6a). Such findings may suggest that there is an increased age-independent differentiation of RTE Tregs via CD31^+^ memory Tregs into CD31^−^ memory Tregs in SLE patients, which ensures the preservation of the Treg pool, while the proliferation capacity of the memory Tregs is severely suppressed by corticosteroids. A proliferation-inhibiting effect of corticosteroids could not be detected for Tresp cells (Fig. 6b).

### Proliferation inhibiting drugs suppress the accelerated age-dependent differentiation of both RTE-Tregs and RTE-Tresps via CD31^+^-memory-Tregs/Tresps

**Fig. 7:**
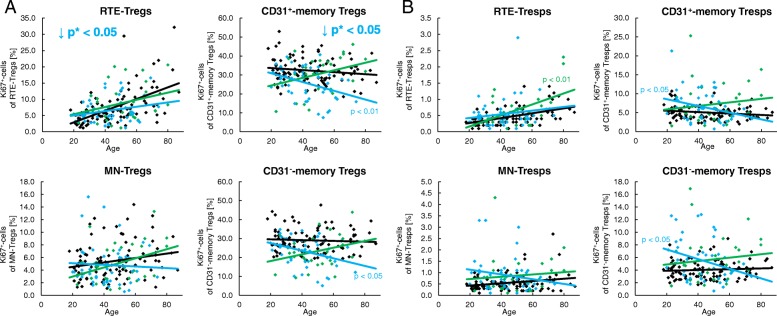
Changes of the Ki67 expression of recent thymic emigrant (RTE), mature naive (MN), CD31^+^, and CD31^−^ memory regulatory T cells (Tregs)/responder T cells (Tresps) with age in healthy volunteers (*n* = 94) and SLE patients who were treated with MMF or AZA (*n* = 48) or not (*n* = 30). The percentages of Ki67^+^ cells within RTE, MN, CD31^+^, and CD31^−^ memory Tregs/Tresp was estimated in healthy volunteers (black diamonds) and in SLE patients who were treated with MMF or AZA (blue diamonds) or not (green diamonds). Significant changes with age are marked by blue *p* values (treated with AZA or MMF) or green *p* values (no AZA or MMF medication). An age-independent significantly decreased (blue downward arrow) percentage of Ki67^+^ cells was detected in RTE Tregs and CD31^+^ memory Tregs (marked by blue p* values) of AZA- or MMF-treated versus untreated SLE patients (**a**). Moreover, in SLE patients who did not receive AZA or MMF the proliferative capacity of RTE Tresps increased significantly with age, while this was not the case when AZA or MMF medication was given to the SLE patients. The proliferation capacity of CD31^+^ and CD31^−^ memory Tresps did not rise significantly with age if the SLE patients were not treated with AZA or MMF, but it decreased significantly if the SLE patients had been treated with AZA or MMF (**b**). Similar results, with a clear decrease in the proliferation capacity of CD31^+^ and CD31^−^ memory Tregs with age and a significant difference in the slopes of the regression lines between treated and untreated SLE patients, were also observed for Tregs (**a**)

Figure 7 shows the proliferation capacity of all four Treg/Tresp subsets depending on whether the patients were treated with either azathioprine (AZA) or mycophenolate mofetil (MMF) (*n* = 48) or not (*n* = 30). Clinically, these two patient groups differed regarding their urine protein/urine creatinine ratio (Table 1). Regardless of age, this medication reduced the proliferation capacity of RTE and CD31^+^ memory Tregs significantly compared with those who had not received these drugs (Fig. 7a). Therefore, these results also emphasize that there is an increased differentiation of RTE Tregs via CD31^+^ memory Tregs into CD31^−^ memory Tregs in SLE patients compared with healthy controls which is inhibited by the immunosuppressive therapy. In contrast to the effect of corticosteroids, there was also an effect of azathioprine or mycophenolate mofetil on the proliferative capacity of Tresps. In SLE patients who did not receive this medication, the proliferative capacity of RTE Tresps increased significantly with age, while this was not the case when azathioprine or mycophenolate mofetil medication was given to the SLE patients. Also, the proliferation capacity of CD31^+^ and CD31^−^ memory Tresps did not rise significantly with age if the SLE patients were not treated with azathioprine or mycophenolate mofetil, but it decreased significantly if the SLE patients had received this medication (Fig. 7b). Astonishingly, similar results with a clear decrease in the proliferation capacity of CD31^+^ and CD31^−^ memory Tregs with age and a significant difference in the slopes of the regression lines (*p* < 0.01) between treated and untreated SLE patients were also observed (Fig. 7a). These findings also suggest that there is an age-dependent increased differentiation of both RTE Tregs and RTE Tresps via CD31^+^ memory Tregs/Tresps into CD31^−^ memory Tregs/Tresps in SLE patients which is strongly suppressed by azathioprine or mycophenolate mofetil medication. This medication seems to be of particular importance since these substances seem to selectively inhibit the accelerated age-dependent differentiation of RTE Tresps via CD31^+^ memory Tresps into CD31^−^ memory Tresps, and at the same time inhibit the differentiation of RTE Tregs via CD31^+^ memory Tregs into CD31^−^ memory Tregs. It is striking that this medication reduces the age-dependent proliferation capacity of the memory Tregs from normal to reduced levels but that of memory Tresps from increased to normal levels.

### SLE patients show an impairment of both Treg and Tresp functionality

**Fig. 8:**
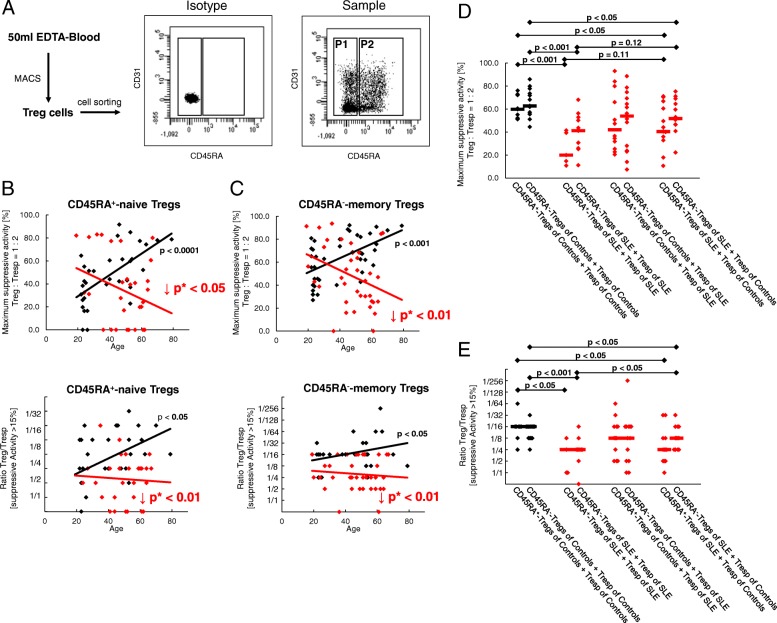
Suppressive activity of naive CD45RA^+^ regulatory T cells (Tregs) and CD45RA^−^ memory Tregs obtained from healthy volunteers (*n* = 40) and systemic lupus erythematosus (SLE) patients (*n* = 37). Total CD4^+^CD127^low+/–^CD25^+^ Tregs were isolated by magnetic-activated cell sorting (MACS), stained with anti-CD45RA and CD45RO monoclonal antibodies and sorted into CD45RA^−^ memory Tregs (P1) and naive CD45RA^+^ Tregs (P2) (**a**). The suppressive activity of both Treg subsets was estimated with autologous Tresp for healthy volunteers (black diamonds) and SLE patients (red diamonds). The figure shows the individual and median values of the maximum suppressive activity (Treg/Tresp = 1/2) and of the ratio of Treg to Tresp up to which the purified CD45RA^+^ and CD45RA^−^ Treg subsets could be diluted to achieve a minimum suppressive activity of at least 15% (**b** and **c**). In healthy volunteers, the suppressive activity of both Treg subsets increased significantly with age. However, this could not be sustained for SLE patients (**b** and **c**). Furthermore, the suppressive activity of both Treg subsets obtained from healthy volunteers (*n* = 14) and SLE patients (*n* = 14) was tested with nonautologous Tresps of age-matched SLE patients and nonautologous Tresps of age-matched healthy volunteers. The suppressive activity of both Treg subsets from SLE patients was significantly reduced when tested with both autologous and nonautologous Tresps of healthy volunteers (**d** and **e**)

To examine whether there were age-related differences in the suppressive activity of separated naive CD45RA^+^ and CD45RA^−^ memory Tregs, the total CD4^+^CD127^low+/–^CD25^+^ Treg pool of 40 healthy volunteers (mean age 41 ± 17 years) and 37 SLE patients (mean age 49 ± 13 years) was isolated by magnetic-activated cell sorting (MACS) and sorted into both Treg subsets (Fig. 8a). Subsequently, the isolated CD45RA^+^ naive Tregs and CD45RA^−^ memory Tregs were analyzed separately for their suppressive capacity. We found that the suppressive activity of both naive CD45RA^+^ and CD45RA^−^ memory Tregs of healthy controls increased significantly with age, while this could not be maintained in SLE patients (Fig. 8b, c). Moreover, regardless of age, the functionality of both Treg subsets was significantly diminished in SLE patients compared with healthy volunteers (Fig. 8b, c).

To further clarify whether the Treg function of SLE patients is diminished or Tresp function is enhanced, we examined the suppressive activity of CD45RA^+^ naive Tregs and CD45RA^−^ memory Tregs purified from 14 middle-aged SLE patients (mean age 49 ± 11 years) with both autologous Tresps and nonautologous Tresps purified from age-matched healthy volunteers (mean age 43 ± 14 years). Furthermore, the suppressive activity of both Treg subsets separated from healthy volunteers was tested with both autologous Tresps and nonautologous Tresps purified from age-matched SLE patients. Figure 8d and e show that the maximum suppressive activity of both naive CD45RA^+^ and CD45RA^−^ memory Tregs of SLE patients was significantly lower than that of healthy volunteers when tested with autologous Tresps. When tested with nonautologous Tresps of healthy volunteers, their suppressive activity was increased; however, statistical significance was not achieved. Nevertheless, the Tregs of healthy volunteers were able to suppress the Tresps of SLE patients as well as their own Tresps. In addition, the suppressive activity of both naive CD45RA^+^ and CD45RA^−^ memory Tregs of SLE patients was significantly lower when tested on nonautologous Tresps of healthy volunteers compared with the suppressive activity of Tregs of healthy volunteers tested on their own Tresps. In summary, our findings suggest that both Tregs and Tresps are functionally disturbed in SLE patients. Thereby, it seems that the diminished sensitivity of their Tresps for the inhibition by their Tregs, which obviously aggravates with age in these patients, is relatively well controlled by the currently used immunosuppressive therapy. However, this therapy may also have the effect of reducing Treg functionality.

## Discussion

It has been known for many years that an increased CD4^+^ T-cell stimulation in SLE patients leads to the accumulation of terminal differentiated effector memory T cells with decreased proliferative capacity and increased apoptosis sensitivity [22–25]. Consequently, a reduction in circulating CD4^+^ T cells was found in these patients [26]. However, until now the particular role of immunosuppressive CD4^+^ Tregs or CD4^+^ effector Tresps in the pathogenesis of SLE remains elusive. An imbalance in the ratio of Tregs to Tresps is thought to be the most important cause of the disturbed T-cell signaling in SLE patients. The mechanisms that trigger this imbalance are currently being investigated more intensively.

In this study, we examined the differentiation of RTE Tregs/Tresps into CD31^−^ memory Tregs/Tresps and verified whether changes in the differentiation pathway via MN Tregs/Tresp or CD31^+^ memory Tregs/Tresps had an effect on the balance between Tregs and Tresps which may affect the functionality of the Treg pool. We found an increased differentiation of RTE Tregs/Tresps into CD31^−^ memory Tregs/Tresps with age in both healthy volunteers and SLE patients. However, regardless of age, SLE patients revealed a greater differentiation of the Treg pool, but did not show differences in the Tresp pool. The Ki67 staining of the different Treg/Tresp cell subsets revealed a significant age-independent reduction in CD31^+^ and CD31^−^ memory Treg proliferation, suggesting a predominant differentiation via CD31^+^ memory Tregs in SLE patients, which most probably is strongly restrained by the immunosuppressive drugs, even below the normal level of healthy volunteers. In contrast, all Tresp subsets showed significantly increased proliferation suggesting an age-independent increased RTE Tresp cell differentiation via both pathways in spite of the immunosuppressive therapy. Our experiences with pregnant women and dialysis and transplant patients suggest that the favored differentiation pathway of RTE Tregs/Tresps via CD31^+^ memory Tregs/Tresps or MN Tregs/Tresps may increase or reduce the apoptosis sensitivity of the arising CD31^−^ memory Tregs/Tresps [14, 17, 27]. Consequently, our data assume that the size of the Treg pool should be largely maintained in SLE patients, while that of the Tresp pool should be diminished due to reduced production and increased consumption of resting naive MN Tresps. Nevertheless, it is expected that the functionality of the Treg pool will be weakened as the proliferation capacity of memory Tregs was found to be reduced below the level of healthy controls.

**Fig. 9:**
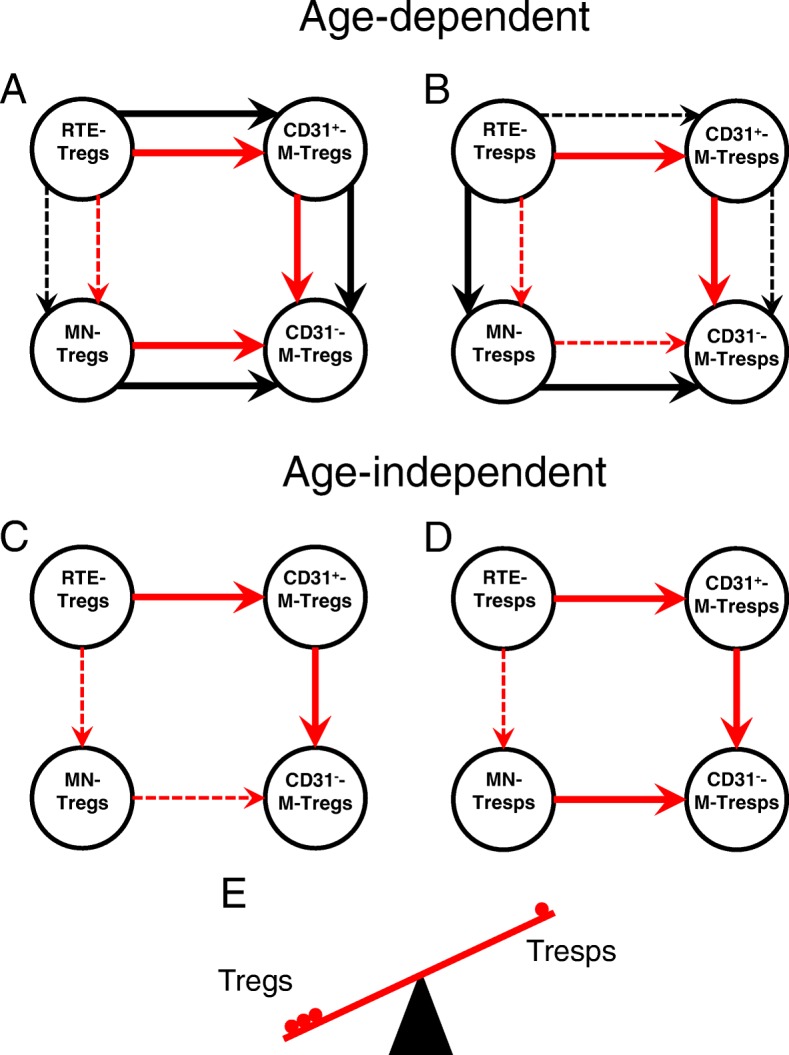
Age-dependent and age-independent differentiation of regulatory T cells (Tregs)/responder T cells (Tresps) in healthy volunteers and SLE patients. The figure summarizes our findings concerning the age-dependent (**a** and **b**) and age-independent (**c** and **d**) differentiation of recent thymic emigrant (RTE) Tregs (**a** and **c**) or RTE Tresps (**b** and **d**) via mature naive (MN) Tregs/Tresps or CD31^+^ memory Tregs/Tresps into CD31^−^ memory Tregs/Tresps for healthy controls (black arrows) and SLE patients (red arrows). An age-dependent increased differentiation of RTE Tregs via CD31^+^ memory Tregs into CD31^−^ memory Tregs, as well as an age-dependent increased conversion of resting naive MN Tregs into CD31^−^ memory Tregs, was found for both healthy controls and SLE patients (**a**, illustrated by red and black bold arrows). In contrast, with increasing age, an opposite differentiation was found for RTE Tresps, which differentiated increasingly via MN Tresp into CD31^−^ memory Tresps in healthy controls, while those of SLE patients differentiated via CD31^+^ memory Tresps into CD31^−^ memory Tresps (**b**, illustrated by black and red bold arrows). An age-dependent increased differentiation of resting naive MN Tresps into CD31^−^ memory Tresps was not found for healthy controls or for SLE patients (**b**). Regarding the age-independent differentiation, our findings show an increased differentiation of RTE Tregs/Tresps via CD31^+^ memory Tregs/Tresps into CD31^−^ memory Tregs/Tresps in SLE patients compared with healthy controls (**c** and **d**, illustrated by red bold arrows). Additionally, we found an age-independent increased conversion of resting naive MN Tresps into CD31^−^ memory Tresps in SLE patients compared with healthy controls (**d**, illustrated by a red bold arrow). These mechanisms may lead to an imbalance between Tregs and Tresps in favor of Tregs (**e**), especially when the pool of these resting naive MN Tresps is no longer filled up

Figure 9 summarizes the different age-dependent and age-independent differentiation pathways of RTE Tregs/Tresps in both healthy controls and SLE patients. It can be assumed that in both the total Treg and the total Tresp pool a population of resting naive MN Tregs/Tresps exists during the course of life [28]. For healthy controls, it appears that RTE Tregs differentiate increasingly via CD31^+^ memory Tregs into CD31^−^ memory Tregs. However, there is also an increased conversion of resting naive MN Tregs into CD31^−^ memory Tregs with age (Fig. 9a). On the other hand, an increased differentiation of RTE Tresps via MN Tresps into CD31^−^ memory Tresps was observed in healthy controls, whereby resting naive MN Tresps were rather enriched within the total CD31^−^ Tresp pool instead of being converted into CD31^−^ memory Tresps (Fig. 9b). For SLE patients, a similar age-dependent differentiation could be revealed for RTE Tregs (Fig. 9a). However, in contrast to healthy controls, RTE Tresps of SLE patients differentiated increasingly via CD31^+^ memory Tresps into CD31^−^ memory Tresps (Fig. 9b). This altered differentiation of RTE Tresps suggests that highly apoptosis-resistant Tresps may arise, particularly in old-aged SLE patients, which reduces the autologous Treg function in these patients. These results are in line with clinical observations that old age-onset SLE is not benign and more disease activity and damage are present in these patients [29]. Such findings are confirmed by our data showing a significant increase of the Treg function with age in healthy volunteers, which cannot be maintained in SLE patients. In addition, our investigations testing the Treg function of SLE patients on both autologous and nonautologous Tresps also show that the suppressive activity of SLE Tregs is indeed reduced when tested with autologous Tresps, but it is better, though not significantly so, when tested with nonautologous Tresps of healthy controls. These findings suggest that the sensitivity of Tresps to the suppressive function of Tregs may be reduced in SLE patients. Similar results were published by Venigalla et al., who also showed impaired Tresp sensitivity to the suppressive function of both autologous and nonautologous Tregs isolated from patients with active SLE, where the functionality of Tregs was fully preserved [30]. These data are in line with results using a mouse lupus model, in which similar crossover experiments revealed an impaired Tresp cell sensitivity in MRL/Mp mice [31]. In contrast, our data reveal that Tregs obtained from SLE patients showed limited functionality when tested with both autologous Tresps and allogeneic Tresps from healthy volunteers, indicating a generally impaired Treg functionality in SLE remission patients.

Beside these differences concerning the age-dependent differentiation of RTE Tregs/Tresps between healthy controls and SLE patients, our data also reveal that there may be a general age-independent increased differentiation of RTE Tregs/Tresps via CD31^+^ memory Tregs/Tresps into CD31^−^ memory Tregs/Tresps in SLE patients compared with healthy controls (Fig. 9c, d). However, in contrast to healthy controls who rather enrich resting naive MN Tresps, there seems to be an increased age-independent conversion and consumption of MN Tresps in SLE patients (Fig. 9d). This mechanism may affect the balance between Tregs and Tresps substantially (Fig. 9e), especially when the pool of these resting naive MN Tresps is no longer filled up. Presumably, this phenomenon may be strongly pronounced in patients with active disease.

It should be considered that, in our study, Tregs and Tresps were exclusively isolated from patients in remission but not from patients with active SLE. Therefore, exaggerated Treg/Tresp cell activation may have been largely restrained by extensive immunosuppression therapy. Therefore, it is striking that azathioprine or mycophenolate mofetil medication specifically and substantially reduced the accelerated age-dependent increase of RTE Tresps differentiation via CD31^+^ memory Tresps into CD31^−^ memory Tresps. A similar effect of this medication was also found for the accelerated age-dependent differentiation of RTE Tregs. However, this reduced the proliferation capacity of the arising memory Tregs far below the normal level of healthy controls, while that of the resulting Tresps was reduced from increased to normal levels. This may be caused by the fact that the initial degree of activation of the RTE Tresps is strongly increased in SLE patients compared with that of healthy volunteers. While the effect of these substances on the Tresps is desired, their effect on Tregs is adverse. Corticosteroid therapy did not have any effect on Tresp differentiation or proliferation. However, it strongly reduced the proliferative capacity of the memory Tregs. A drug-induced reduction in Treg functionality due to diminished proliferation capacity of the memory Tregs could therefore be observed in SLE remission patients and be the cause for ongoing discussions regarding the effectiveness of corticoid treatment [32], although it was shown to cause Treg cell expansion [33, 34].

Current discrepancies concerning the number and function of Tregs in SLE patients will probably mainly arise from the fact that different collectives regarding the activation status of the patients were examined. It seems quite clear that decreased Treg cell counts and reduced functionality were mainly found in patients with high clinical disease activity [35, 36]. It still remains unclear whether or which specific Treg subsets are affected and to what extent the differentiation or stability of individual Treg subsets is impaired. More selective agents that have less negative influence on Treg differentiation would be desirable, and rapamycin is increasingly coming into focus. While this substance does not yet play a role in the treatment of SLE patients, there is increasing clinical evidence of potentially good efficacy in SLE patients [37]. Its mechanistic target is mTOR, an evolutionally conserved kinase consisting of two interacting complexes (mTORC1 and mTORC2) that regulate T-cell lineage specification and macrophage differentiation [38]. It was shown that mTOR is highly activated in Tregs of SLE patients and that mTORC1 and mTORC2 were stimulated by IL-21, by which the differentiation and function of the Treg cells was abrogated. Rapamycin treatment restored Treg function [39], indicating that mTOR signaling in Tregs constitutes a potential checkpoint in lupus pathogenesis. Moreover, mTOR signaling also controls the differentiation of multiple effector T cells, including Th1, Th2, Th17, and particularly that of follicular T-helper (Tfh) cells [40, 41]. Such Tfh cells are known to provide optimal B cell-mediated humoral immunity and were shown to be involved in the pathogenesis of SLE, whereby the ratio of circulating Tfh to follicular T regulatory (Tfr) cells is correlated with disease activity in SLE [42, 43]. Another interesting therapeutic effect of rapamycin is its ability to reverse the senescent phenotype of mesenchymal stem cells from MRL/lpr mice and SLE patients through inhibition of the mTOR signaling pathway [44], suggesting that cellular senescence of cells constituting the microenvironment of hematopoietic stem cells may play an important role in the pathogenesis of SLE. Further studies confirm that such immunosuppressive mesenchymal stem cells show characteristic signs of senescence [45–47] and therefore suggest that allogeneic rather than autologous mesenchymal stem cell transplantation may be promising in the treatment of drug-resistant SLE [48].

## Conclusions

In summary, our data reveal that there is a potential effect of the currently used therapeutics especially on the age-dependent accelerated T-cell differentiation in SLE patients. Therefore, it seems likely that the disruption of immunosuppression in active SLE causes severe differentiation, senescence, and depletion of T cells, whereby the favorable ratio of Tregs/Tresps in SLE remission patients may be changed in favor of Tresps. The identification of the special Treg/Tresp subsets involved in these mechanisms may be helpful for further elucidation of the pathogenesis of SLE and for development of effective therapeutic strategies in the treatment of SLE.

## Additional file

Additional file 1: **Figure S1.** Changes in the composition of the total CD4^+^ T-helper cell pool with age in healthy volunteers (*n* = 94) and SLE patients (*n* = 78). The percentages of RTE Tregs/Tresps, MN Tregs/Tresps, CD31^+^ memory Tregs/Tresps, and CD31^−^ memory Tregs/Tresps were estimated within the total CD4^+^ T-helper cell pool in both healthy volunteers (black diamond) and SLE patients (red diamond). The figures present the regression lines concerning the changes in the percentages of the different Treg/Tresp subsets with increasing age. Significant changes with age are marked by black *p* values (healthy volunteers) or red *p* values (SLE patients). Significantly increased percentages (red upward arrow) of MN, CD31^+^ memory Tregs, and CD31^−^ memory Tregs independently of age (marked by red p* values) suggest a differentiation which rather increases Tregs (A) than Tresps (B) in SLE patients compared with healthy volunteers. (PPTX 273 kb)

## Abbreviations

ACR: American College of Rheumatology
AZA: Azathioprine
CD31^+^ memory Tregs/Tresps: CD45RA^−^CD31^+^ memory Tregs/Tresps
CD31^−^ memory Tregs/Tresps: CD45RA^−^CD31^−^ memory Tregs/Tresps
FACS: Fluorescence-activated cell sorting
FSC: Forward-scatter characteristics
IL: Interleukin
MACS: Magnetic-activated cell sorting
MMF: Mycophenolate mofetil
MN: Mature naive
PBMC: Peripheral blood mononuclear cell
RTE: Recent thymic emigrant
SLE: Systemic lupus erythematous
SLEDAI: Systemic Lupus Erythematous Disease Activity Index
SSC: Side-scatter characteristics
Tfh: Follicular T-helper
Tfr: Follicular T-regulatory
Th: T-helper
Treg: Regulatory T cell
Tresp: Responder T cell
