# Supplementary material for: Immunosuppressive therapy influences the accelerated age-dependent T-helper cell differentiation in systemic lupus erythematosus remission patients
Source: Arthritis Res Ther. 2018 Dec 18;20:278. doi: 10.1186/s13075-018-1778-6 (PMC6299578; doi:10.1186/s13075-018-1778-6)
Supplement: Supplementary file 1 — Figure S1. Changes in the composition of the total CD4+ T-helper cell pool with age in healthy volunteers (n = 94) and SLE patients (n = 78). The percentages of RTE Tregs/Tresps, MN Tregs/Tresps, CD31+ memory Tregs/Tresps, and CD31− memory Tregs/Tresps were estimated within the total CD4+ T-helper cell pool in both healthy volunteers (black diamond) and SLE patients (red diamond). The figures present the regression lines concerning the changes in the percentages of the different Treg/Tresp subsets with increasing age. Significant changes with age are marked by black p values (healthy volunteers) or red p values (SLE patients). Significantly increased percentages (red upward arrow) of MN, CD31+ memory Tregs, and CD31− memory Tregs independently of age (marked by red p* values) suggest a differentiation which rather increases Tregs (A) than Tresps (B) in SLE patients compared with healthy volunteers. (PPTX 273 kb) [file 13075_2018_1778_MOESM1_ESM.pptx]

## Slide 1
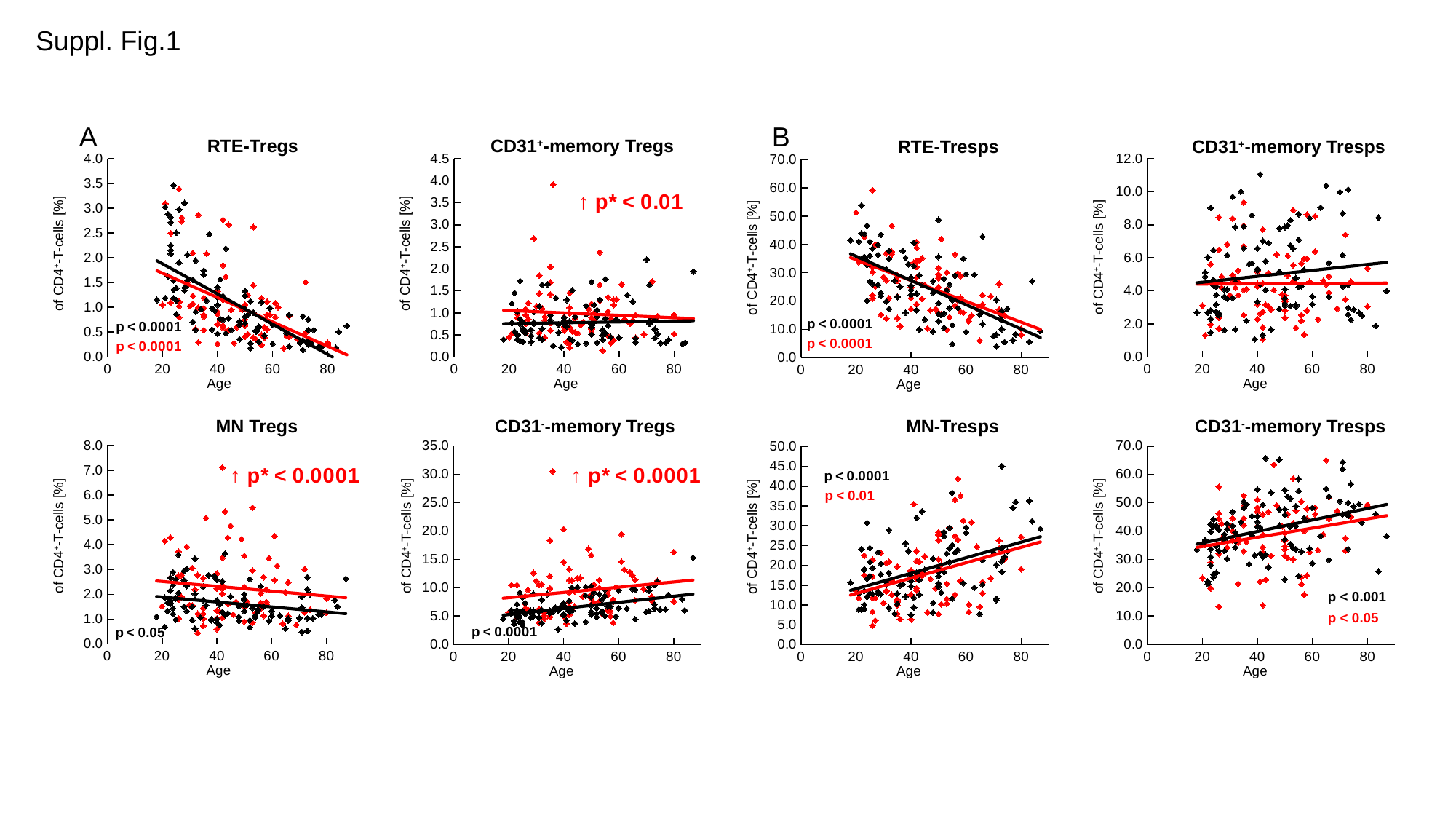

Suppl. Fig.1
A
B
CD31+-memory Tregs
RTE-Tregs
CD31+-memory Tresps
RTE-Tresps
### Chart
| Category | RTEs | SLE |
|---|---|---|
### Chart
| Category | | |
|---|---|---|
### Chart
| Category | | |
|---|---|---|
### Chart
| Category | RTEs | Spalte1 |
|---|---|---| of CD4+-T-cells [%]
 of CD4+-T-cells [%]
 of CD4+-T-cells [%]
 of CD4+-T-cells [%]
MN-Tresps
MN Tregs
CD31--memory Tregs
CD31--memory Tresps
### Chart
| Category | MNs | Spalte1 |
|---|---|---|
### Chart
| Category | CD31-Mem | Spalte1 |
|---|---|---|
### Chart
| Category | CD31-Mem | Spalte1 |
|---|---|---|
### Chart
| Category | MNs | Spalte1 |
|---|---|---| of CD4+-T-cells [%]
 of CD4+-T-cells [%]
 of CD4+-T-cells [%]
 of CD4+-T-cells [%]
p < 0.001
p < 0.05
